# Observing mother-child interaction in a free-play vs. a structured task context and its relationship with preterm and term born toddlers' psychosocial outcomes

**DOI:** 10.3389/frcha.2023.1176560

**Published:** 2023-09-07

**Authors:** L. J. G Krijnen, M. Verhoeven, A. L. van Baar

**Affiliations:** Child and Adolescent Studies, Utrecht University, Utrecht, Netherlands

**Keywords:** observational context, mother-child interaction, psychosocial outcomes, structured task, free-play, moderate to late preterm, social-emotional, internalizing and externalizing behaviors

## Abstract

**Introduction:**

High quality of mother-child interaction is associated with better psychosocial outcomes in children. However, this association might depend on the context in which mother-child interaction is observed as well as specific child characteristics. In this study, we examine differences in the assessment of mother-child interaction in a free-play and a structured task context. In addition, it will be investigated whether the behaviors per context are differently associated with preterm vs. term born toddlers' psychosocial outcomes.

**Methods:**

A total of 201 Dutch mother-child dyads participated in the study, of whom 108 children were moderate to late preterm (MLP) and 93 were born at term. Mother-child interaction was observed in a free-play and a structured task context when the child was 18 months of (corrected) age. Six subscales of mother-child interaction were assessed using the Coding Interactive Behavior scheme: maternal stimulation, maternal warmth, child's negative affect, active mother and child engagement, dyadic synchrony and tense interaction. Psychosocial outcomes were assessed at 24 months of (corrected) age using the Ages and Stages Questionnaire – Social Emotional and the Child Behavior Checklist.

**Results:**

Mother-child interaction was reliably assessed (α > .60) in each context, except for tense interaction during free-play (α = .41) and child's negative affect when averaged across contexts (α = 0.55). Compared to the free-play context, during the structured task, more child's negative affect, tense interaction and active mother and child engagement was observed in MLP and term born children, and less dyadic synchrony in MLP children (*p*'s < .01). Only during a structured task and for term born children, active mother and child engagement was related to less social-emotional difficulties, internalizing and externalizing behaviors. Only during free-play and for MLP children, active mother and child engagement was related to less externalizing behaviors. Dyadic synchrony during a structured task was associated with less social-emotional difficulties in MLP and term born children, and dyadic synchrony during free-play was only associated with less social-emotional difficulties in term born children (all *p*'s < .05).

**Discussion:**

Most mother-child interactive behaviors can be reliably assessed in both contexts. The structured task context elicited more varied behaviors than the free-play context. With the observations in the structured task context, more associations with children's psychosocial outcomes were found than with the observations in the free-play context. Mother-child interactions characterized by active, engaged and synchronous behaviors were associated with better psychosocial outcomes in toddlers, with some differences observed for MLP vs. term born children and for the free-play vs. the structured task context. Suggestions for future research as well as clinical practice are provided.

## 1. Introduction

According to the ecological system theory of Bronfenbrenner, child development is partially shaped by its environment (1). The interaction between mother and child is one of the core aspects of a child's direct environment, in particular during the first years of life. A high quality of mother-child interaction – characterized by fluent, warm and reciprocal interactions – has found to be associated with positive psychosocial outcomes, such as better social-emotional skills, and less internalizing and externalizing problems in the children (2–5). Mother-child interaction is usually assessed in a free-play and/or a structured task context, which may affect the behaviors mothers and children show during the observation (6, 7). Moreover, associations between interactive behaviors and children's psychosocial outcomes may depend on the context (8, 9) and on whether the child is at risk for psychosocial problems, like preterm born children (2, 10).

Some studies use both contexts to observe mother-child interaction (e.g. 8, 11), whereas other studies choose one or the other context (e.g. 2, 12). In a free-play context, mother and child can play together in their own preferred way, often freely choosing out of a selection of toys, whereas in a structured task context, mother and child are instructed to work on a specific task together (e.g., making a puzzle). The context in which mother-child interaction is observed can lead to different conclusions about the quality of the interaction and its association with children's psychosocial outcomes due to the different types of behaviors that may be elicited. It is therefore important to gain more knowledge regarding the role of context in observing mother-child interactions and its relationship with children's psychosocial outcomes.

 Several studies on mothers and their 0–5 years old children have compared behaviors between the two contexts. The most consistent finding is that mothers were more intrusive in structured task contexts than in free-play contexts (6, 7, 13, 14). Another relatively consistent finding is more positive behaviors, e.g., engagement, positive affect, sensitivity and responsiveness, during free-play compared to structured task contexts in parents (6, 11, 13, 14) and children (6, 7, 14). However, findings of a study by Volling et al. (2002) slightly differ from this: like previous studies, they found that children (12 months) showed more positive behavior during the free-play context, but the mothers of these children in contrast showed more positive behavior during the structured task. For older children (5–12 years old), Dittrich et al. (2017) found higher levels of positive behaviors in children and their mothers during a structured task and speculate that more supportive and responsive behaviors of mothers and children is elicited by setting a mutual goal – in this case making a puzzle – which may induce “positive stress” and thereby increases functional behaviors. However, their finding might be an age-specific result considering the older age range in the study of Dittrich et al. (2017) and the contradiction with other studies in younger children. Even though findings of previous studies slightly differ, there seems to be consensus that the behaviors observed are dependent on the observational context.

The observational context seems to affect the associations between mother-child interaction and children's outcomes. Focusing on maternal behaviors only, Nordahl et al. (2020) found that parenting quality during the semi-structured task context – i.e., shape sorting blocks and making a puzzle – was more predictive for children's outcomes than parenting quality during the free-play context. Even though parenting quality was not compared between both contexts, the results indicate that the semi-structured task context elicits more meaningful maternal behaviors in predicting children's outcomes. Potentially, certain behaviors are more elicited under certain circumstances, e.g., structured task, and are therefore found to be related to children's outcomes. Dittrich et al. (2017) did compare mother-child interactive behaviors between the contexts and found that children's responsiveness was higher in a structured task context, and more importantly, only within this structured context more child responsiveness was associated with less parent-reported externalizing problems. However, Dittrich et al. (2017) also found evidence that it is not necessarily the extent to which behaviors are shown that matter. Rather, the circumstances in which the behavior is shown are important. To illustrate, maternal emotional availability was higher during the structured task, but the relationship between maternal emotional availability and children's problem behavior was significantly stronger for the free-play context than the structured task context. This indicates that the seemingly same behavior, e.g., maternal emotional availability, may be more important to be present under certain circumstances, e.g., free-play, than in other circumstances, e.g., structured task. However, it is unclear why this is the case and more research is needed to confirm these findings and identify reasons for this effect (8).

Not only the context but also child characteristics, such as preterm birth, may play an important role in the association between mother-child interaction and children's psychosocial outcomes. Krijnen et al. (2022) – who used the same sample as the current study – found that certain mother-child interactive behaviors – i.e., mother-led interaction, reciprocal engagement – were positively related with children's psychosocial outcomes when children were born at term, but not when children were born moderately to late preterm [MLP, 32–37 weeks gestational age (GA)]. So, it seems that only for term born children, but not for MLP children, certain interactive behaviors were related to better psychosocial outcomes. Krijnen et al. (2022) used a structured task context but there is evidence that results are different for a free-play context in a sample of very preterm born children (<33 weeks of GA). Gueron-Sela et al. (2015) observed both parents with their 6 month old infant in a free-play context and reported different results: When the quality of parent-child interaction was high and parental stress was low, preterm children outperformed their term born peers with respect to their social competences at 12 months of age. If the circumstances were reversed, i.e., high stress and low quality of interaction, preterm children had worse social competences than their term born counterparts (10). Landry and colleagues (15) found similar results: If parents were responsive to their child in the first year of life – as observed during free-play and daily activities, e.g., bathing – preterm children showed more growth in terms of their social and emotional competences than term born children. Based on these studies, it seems that the association between mother-child interaction and children's psychosocial outcomes may differ for children born preterm vs. at term, and that inconsistencies in findings might result from observations in different contexts.

The existing body of literature indicates that the relationship between mother-child interaction behaviors and psychosocial outcomes depend on 1) the observational context in which interactions are observed, and 2) the birth status of the child. However, no direct comparisons between a free-play and a structured task context in relation to MLP and term born toddlers' psychosocial outcomes have been made yet. The current study explores which interactive behaviors within a free-play and within a structured task context are associated with psychosocial outcomes in MLP and term born toddlers. To capture a broad picture of the child's psychosocial outcomes, three psychosocial outcome measures will be assessed: social-emotional difficulties, internalizing behaviors and externalizing behaviors. Three objectives will be addressed, with the first one being the basic evaluation of whether both observational contexts are suited to reliably assess mother-child interaction. Subsequently, interactive behaviors will be compared between observational contexts and between MLP and term born children. It is hypothesized that during the free-play context more positive behaviors – e.g., positive affect, engaged behaviors – are shown compared to the structured task context. Third, for each context will be explored whether mother-child interaction behaviors at 18 months (corrected) age are associated with psychosocial outcomes at 24 months (corrected) age and if these relationships are significantly different for MLP and term born children. No hypothesis was formulated due to the limited research on examining the role of observational context in predicting psychosocial outcomes for MLP vs. term born children and the current research is therefore of exploratory nature. With the current study, more knowledge will be obtained regarding the role of the observational context in assessing mother-child interaction and its relationship with children's psychosocial outcomes. Hence, recommendations can be provided for both researchers and clinicians about the preferred context for observing mother-child interaction, based on their specific goals (e.g., elicit certain behaviors, predict psychosocial outcomes in term born or preterm born children). Furthermore, insight will be gained in which interactive behaviors in which context are associated with better psychosocial outcomes in preterm vs. term born children.

## 2. Methods

### 2.1. Participants

The current study forms part of a larger Dutch project called Study on Attention of Preterm children (STAP) in which MLP and term born children were longitudinally assessed. Children were recruited by midwives and pediatricians from nine hospitals around Utrecht, the Netherlands, at 10 months of age, between March 2010 and April 2011. Exclusion criteria were admission to a tertiary Neonatal Intensive Care Unit (NICU), severe congenital malformations, multiple births, dysmaturity – i.e., a birthweight below the 10th percentile of the weight expected for infants' gestational age using Dutch reference curves (16) - , maternal antenatal substance abuse or chronic antenatal use of psychiatric drugs. Both parents, or one parent in the case of single parent families, had to sign an informed consent in order to participate in the study.

Initially, the sample consisted of 226 participants. Participants were included for the current study if 1) mother-child interaction was observed at 18 months of (corrected) age, and 2) at least one of the three psychosocial outcomes measures was completed at 24 months of (corrected) age. This led to a final sample of 201 participants, of whom 108 MLP and 93 term born children. See Table 1 for the characteristics of the participants.

**Table 1:** Participant characteristics per group of birth status.

	MLP (*n* = 108)	Term (*n* = 93)
Gender		
Male (*n*, %)	63 (58.33%)*	41 (44.09%)
Female (*n*, %)	45 (41.67%)*	52 (55.91%)
Corrected age in months, wave 1		
Mean (SD)	17.22 (0.44)	17.31 (0.47)
Range	17–19	17–18
Corrected age in months, wave 2		
Mean (SD)	23.32 (0.54)	23.59 (0.63)
Range	23–25	23–26
Ethnicity		
Dutch (*n*, %)	104 (96.30%)	89 (96.30%)
Gestational age		
Mean (SD)	34.69 (1.34)***	39.47 (0.98)
Range	32–36	37–41
Birth weight in grams,		
Mean (SD)	2,584.77 (502.21)***	3,576.39 (460.71)
Range	1,420–3,850	2,795–5,330
Education level mother^a^		
Low, *n* (%)	7 (6.48%)	2 (2.15%)
Medium, *n* (%)	36 (33.33%)***	10 (10.75%)
High, *n* (%)	65 (60.19%)***	81 (87.10%)

MLP, Moderate to late prematurely born children; SD, Standard Deviation. To test for group differences, independent samples *t*-tests and Fisher's exact tests were used.

^a^ Low = no education, elementary school, special education, lower general secondary education; Medium = secondary or vocational education; High = college, university or higher.

* *p* < .05.

** *p* < .01.

*** *p* < .001.

### 2.2. Procedure

The STAP study has been approved by the Utrecht Medical Center Ethics Committee (identification code NL34143.041.10). Mother and child were invited for a lab visit at Utrecht University when the child was 18 months of (corrected) age. Appointments were scheduled in such a way that the child's sleeping routine was not disrupted. First, the procedure was explained and then the children's attention capacities were assessed using the Utrecht Tasks of Attention in Toddlers using Eye tracking [UTATE; see (17) for more information] (18 min), which was not used for the current study. After this, mother-child interaction was observed (15 min). Mother-child interaction was observed in two contexts: free-play (5 min) and structured tasks (2 × 5 min). Observations were done in a standardized room, with on one side a play mat with toys, and on the other side a chair and table with a book and a puzzle. Mother-child observations were piloted prior to the data collection to ensure feasibility of the assessment. Free-play was chosen to observe first as this context is a relatively stress-free condition allowing mother and child to adjust to the setting. In the free-play context, the mother was instructed to play with her child as she would normally do at home. Mother and child were sitting on the play mat on the floor, surrounded by a selection of age-appropriate toys (i.e., shape sorter, building blocks, and a pop-up toy). After five minutes, the mother was asked to read a 100-pictures book to her child. Five minutes later, the mother was asked to make a wooden insert puzzle consisting of 11 animal pieces with her child. Both the book and puzzle were selected based on the zone of proximal development for 18 months old children, to ensure the tasks were challenging but not to such an extent that it would cause frustration for the child. After the observation, children received a small present and travel costs were refunded.

All observations were videotaped and coded afterwards for each context separately, resulting in two scores for each rated behavior: one for free-play and one for the structured task context. Two different raters scored the behaviors of the mother-child dyad: one rater scored maternal behaviors and another rater scored child and dyadic behaviors. Raters consisted of students that were trained by our prime trainer, who was qualified as a certified trainer by the developers of the coding scheme that was used (Coding Interactive Behavior scheme; CIB; Feldman, 1998). Following the same procedure as the certified training of Feldman, students had to code practice videos and one final, “golden-standard”, video to assess their reliability. If their scoring was not yet reliable, additional training sessions were provided. When their scoring was reliable, the students started with coding the videos of the current study. As students were instructed to first watch the complete video of a mother-child dyad, and then score a maximum of five behaviors, they had to watch the video multiple times in order to rate all the behaviors. The inter-rater reliability was good, based on 21% double coded videos (ICC = 0.76).

At 24 months of (corrected) age, mothers filled out paper and pencil questionnaires regarding the psychosocial functioning of the child.

### 2.3. Materials

#### 2.3.1. Mother-child interaction – 18 months

Mother-child interaction was scored using the Coding Interactive Behavior scheme (CIB; 18). The CIB assesses mother-child interaction of children aged 2–36 months by scoring behaviors of the parent (21 items; e.g., supportive presence), the child (16 items; e.g., positive affect), and their dyadic interaction (5 items; e.g., fluency of their interaction). Each behavior can be scored on a 5-point Likert scale, ranging from 1 (minimal level of the specific behavior) to 5 (maximal level). The CIB is a globally used tool, that has been well-validated and shows good psychometric properties (18).

The CIB does not have predetermined subscales and, as according to Feldman (19), it depends on the children's age and the cultural background which behaviors fit best in which subscales. Studies therefore created subscales for their own sample. Previous studies used factor analyses to form subscales for the CIB (2, 12, 19), whereas other studies did not describe the method used to form subscales (20–23). For the current study, we carried out an exploratory factor analysis to create subscales that best represented the mother-child interaction behaviors observed in the current sample. To find a factor structure that fitted both contexts and both groups of birth status, the factor analysis was run across contexts and for the total sample. Therefore, before the factor analysis was run, the interactive behaviors were averaged over the contexts and across the groups. Prior to the factor analysis, as a first step, correlations among the behaviors were calculated as Field and colleagues (24) recommend to remove variables showing correlations that are too high (>.80) or too low (<.30). As a second step, KMO and Bartlett's test were calculated to check whether the data and the correlations among the variables were suited for performing an exploratory factor analysis, which was the case (KMO; overall MSA = 0.76, Bartlett's test *p *< .001). Hence, the number of factors was explored based on eigenvalues, scree test and parallel analysis, indicating that a 5 or 6 factor solution suited the data in our study best. Exploratory factor analyses were carried out for the 5 and 6 factor solution, using an oblique rotation method (i.e., oblimin), allowing factors to correlate. Fit indices of both models, i.e., the 5 and 6 factor models, were compared. In addition, the content of the factors was evaluated to determine whether the factors made theoretical sense. Based on these criteria, i.e., the fit indices and the content of the factors, the 6 factor solution was selected (Fit based upon diagonal values = 0.96, RMSEA = 0.07, TLI = 0.81, RMSR = 0.04, SRMR = 0.06). The behaviors included in each factor and the factor loadings can be found in Table 2. All correlations between the factors were small (i.e., between .01 and .29), except for one correlation – between tense interaction and child's negative affect – that was of moderate strength, i.e., .44. This indicates that the factors represent separate aspects of mother-child interaction.

**Table 2:** The definition of the subscales of mother-child interaction and the factor loadings on the subscale.

Subscale	Behaviors (factor loadings)
1. Maternal stimulation	Maternal on-task persistence (.91), maternal resourcefulness (.82), maternal limit setting (.72), maternal elaboration (.70)
2. Maternal warmth	Maternal supportive presence (.84), maternal positive affect (.85), maternal acknowledgement (.69), maternal appropriate variation in affect (.51), maternal negative affect (reversed) (-.48), maternal vocal clarity (.38)
3. Child's negative affect	Child's negative emotionality (.97), child's positive affect (reversed) (-.73), child's labile affect (.69)
4. Dyadic synchrony	Dyadic reciprocity (.78), dyadic adaptation-regulation (.76), dyadic fluency (.38)
5. Active mother and child engagement	Child's reliance on parent for help (.82), child's affection to parent (.80), child's joint attention (.55), child's initiation (.46), mother-led interaction (.35), maternal intrusiveness (.32)
6. Tense interaction	Dyadic tension (.67), dyadic constriction (.53), child's avoidance of parent (.47)

Instead of factor scores, mean scores were used for the analyses. Therefore, we use the term “subscales” instead of “factors” to refer to the types of mother-child interaction. Mean scores were used instead of factor scores for two reasons: 1) factor scores were based on the interactive behaviors across contexts whereas we needed scores per context, 2) using mean scores allows other researchers that have a comparable sample to replicate our results whereas factor scores are dependent on the sample. Mean scores for the subscales were calculated per context of free-play and structured task, by summing the scores of the behaviors and dividing these by the number of scored behaviors. Mean scores could range between 1 and 5, with higher scores showing the behaviors of the subscale more clearly.

#### 2.3.2. Social-emotional difficulties – 24 months

The Ages and Stages Questionnaire – Social Emotional (ASQ-SE; 25) was used to assess social-emotional difficulties at 24 months of (corrected) age. The ASQ-SE measures social-emotional competencies as well as difficulties, by assessing the following dimensions: interaction with people, self-regulation, social-communication, autonomy, adaptive functioning, affect, and compliance. The 24 months age version of the ASQ-SE was used for the current study, which consists of 26 scored items (e.g., “Does your child cry, scream or have tantrums for longer periods of time?” or “Does your child like to be hugged or cuddled?”). Mothers answered whether the child showed the described behavior “most of the time” (0 points), “sometimes” (5 points), and “rarely/never” (10 points). In addition to these 3 answer options, parents could express concerns about the child's behavior for every item, leading to an additional 5 points. Sum scores were calculated, with a higher score reflecting more social-emotional difficulties. Internal consistency of the 24 months version has shown to be good, α = .80 (26). The Dutch version of the ASQ-SE shows adequate specificity (27, 28) and acceptable (28) to slightly insufficient sensitivity (27).

#### 2.3.3. Internalizing and externalizing behavior – 24 months

The Child Behavior Checklist 1½-5 (CBCL; 29) was used to assess internalizing and externalizing behavior. The CBCL is a parent-report questionnaire aiming to assess the child's problem behavior over the past 2 months. The two broadband scales of internalizing and externalizing behavior were used for the current study. The internalizing domain consists of 36 items measuring emotionally reactive behavior (“shows panic for no good reason”), anxious/depressed moods (“too fearful or anxious”), somatic complaints (“nausea, feels sick without medical cause”), and withdrawn behavior (“seems unresponsive to affection”). The externalizing domain consists of 24 items measuring attention problems (“can”t sit still, restless, or hyperactive”), and aggressive behavior (“hits others”). Answer options were “0 = not true”, “1 = somewhat or sometimes true” and “2 = very true of often true”. Sum scores were calculated by adding the answers of the items and standardized T scores were used. Higher scores indicated more internalizing and externalizing behaviors. Validity and reliability of the CBCL 1.5–5 have proven to be good (29). Internal consistency was good for both the internalizing scale (α = .89) and the externalizing scale (α = .92) (30).

### 2.4. Statistical analyses

R version 4.0.3 and SPSS Statistics version 28.0.1.1 were used to analyze the data. First, analyses were executed to investigate the internal consistency of the mother-child interaction subscales that resulted from the exploratory factor analysis. Cronbach's alpha was calculated for each subscale per context and across contexts and values of α above .60 were considered to be sufficient (31). Secondly, mother-child interaction subscales were compared between the free-play and the structured task context per MLP and term born group, and each context was compared between the MLP and term born group, with a repeated measures MANOVA using SPSS. Context was added as a within-subject factor, with two levels (1 = free-play and 2 = structured task). Each mother-child interaction subscale was added as a within-subjects variable per context, resulting in 6 within-subject variables with 2 levels each. Group (0 = term, 1 = MLP) was added as a between-subjects factor. Child's gender (0 = male, 1 = female) and maternal educational level (three dummy variables – i.e., high, medium, low – with low education as the reference category) were added as covariates. Low educational level referred to no education, elementary school, special education, lower general secondary education. Medium educational level referred to secondary or vocational education, and high educational level referred to college, university or higher. Bonferroni correction for multiple testing was applied. Third, multiple regression analyses were run in R to investigate whether mother-child interaction predicted children's psychosocial outcomes – i.e., social-emotional difficulties, internalizing and externalizing behavior. Regression analyses were run separately for the free-play and the structured task context, per outcome measure of psychosocial outcomes, resulting in 2*3 = 6 models. All six mother-child interaction subscales were added as independent variables and were centered prior to the analyses. Group (0 = term born, 1 = MLP born) was added as a dichotomous moderator to investigate whether the relationship between mother-child interaction and children's psychosocial outcomes differed for MLP and term born children. Child's gender and maternal educational level were added as covariates using the same coding as for the repeated measures MANOVA.

## 3. Results

### 3.1. Reliability of the mother-child interaction subscales

Reliability analyses were performed to calculate the Cronbach's alpha for every subscale of mother-child interaction per context and across contexts (See Table 3). All reliability coefficients were acceptable to good (α > .60), except for tense interaction during free-play (α = .41) and child's negative affect across contexts (α = .55).

**Table 3:** Internal consistency of the mother-child interaction subscales.

Subscale	Cronbach's alpha		
	Across contexts	Free-play	Structured task
1. Maternal stimulation	.87	.83	.81
2. Maternal warmth	.75	.75	.75
3. Child's negative affect	.55	.76	.88
4. Dyadic synchrony	.75	.62	.81
5. Active mother & child engagement	.71	.65	.64
6. Tense interaction	.76	.41	.82

### 3.2. Mother-child interaction and psychosocial outcome measures

In Table 4, scores on the mother-child interaction subscales are shown per context (i.e., free-play and structured task), per group (i.e., MLP and term). Psychosocial outcome measures are shown per group.

**Table 4:** Scores on the mother-child interaction subscales per observational context and per MLP and term born group, and the psychosocial outcome scores.

	MLP (*n* = 108)		Term (*n* = 93)	
	Free-play	Structured task	Free-play	Structured task
Mother-child interaction				
Maternal stimulation				
M(SD)	3.72 (0.89)	3.71 (0.84)	3.86 (0.83)	3.88 (0.79)
Range	1.75–5.00	1.50–5.00	2.00–5.00	2.00–5.00
Maternal warmth				
M(SD)	4.77 (0.35)	4.79 (0.35)	4.80 (0.38)	4.83 (0.36)
Range	3.50–5.00	3.17–5.00	3.33–5.00	3.33–5.00
Childs’ negative affect	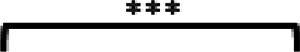		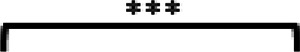	
M(SD)	1.10 (0.27)	1.34 (0.58)	1.06 (0.25)	1.35 (0.63)
Range	1.00–2.33	1.00–3. 67	1.00–2.33	1.00–4.33
Dyadic synchrony	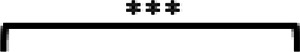			
M(SD)	4.24 (0.68)	3.99 (0.84)	4.23 (0.67)	4.19 (0.74)
Range	2.33–5.00	2.00–5.00	2.66–5.00	1.67–5.00
Active mother and child engagement	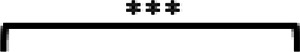		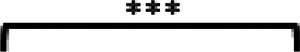	
M(SD)	2.28 (0.63)	2.67 (0.64)	2.31 (0.54)	2.83 (0.63)
Range	1.00–3.67	1.17–4.33	1.17–3.33	1.50–4.25
Tense interaction	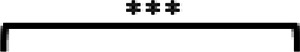		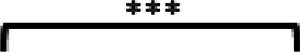	
M(SD)	1.07 (0.22)	1.28 (0.58)	1.05 (0.17)	1.22 (0.45)
Range	1.00–2.33	1.00–3.67	1.00–2.00	1.00–3.33
Psychosocial outcomes				
Social-emotional difficulties^a^, M(SD)	18.17 (11.99)		15.39 (12.45)	
Internalizing behavior^b^, M(SD)	44.76 (8.85)		41.10 (8.11)**	
Externalizing behavior^b^, M(SD)	48.87 (7.96)		46.69 (8.76)	

MLP, Moderate to late preterm; M, mean score; SD, Standard Deviation.

^a^ Data of one MLP child was missing.

^b^ Data of one MLP and two term born children were missing.

* *p *< .05.

** *p *< .01.

*** *p *< .001.

Results of the repeated measures MANOVA revealed differences in mother-child interaction subscales between the free-play and structured task context (see Table 4): Three out of the six mother-child interaction subscales were observed significantly more during the structured tasks than during free-play within both the MLP and the term born group: child's negative affect, active mother and child engagement and tense interaction. In the MLP group, dyadic synchrony was lower during the structured task than during free-play. During free-play, scores on child's negative affect and tense interaction were low, i.e., close to 1, and these variables showed the lowest standard deviation of all the subscales (SD ranging from 0.17 to 0.27), indicating little variation in the scores.

When comparing the MLP and term born group on the mother-child interaction subscales per context, no differences between groups were found.

The current sample scored on average 16.88 (*SD* = 12.25) on social-emotional problems at the ASQ-SE, with scores ranging from 0 to 65. On average, children scored 43.08 (*SD* = 8.69) on internalizing behaviors and 47.87 (*SD* = 8.39) on externalizing behaviors of the CBCL with scores ranging from 28 to 71. The MLP group showed significantly more internalizing behaviors than the term born group [*t*(194.9) = −3.03, *p *= .003]. See Table 4 for scores on the psychosocial outcomes per MLP and term born group.

### 3.3. Regression results of mother-child interaction subscales on psychosocial outcomes

Results of the regression analyses are presented per observational context of free-play and structured task. Per observational context, three separate regression analyses were run per psychosocial outcome measure, i.e., social-emotional difficulties, internalizing and externalizing behaviors. Tables 5, 6 display the results of the three regression analyses for the free-play context and the three regression analyses for the structured task context respectively.

**Table 5:** Results of the regression analyses of mother-child behavior observed in the free-play context, per psychosocial outcome measure.

Free-play context	Social emotional difficulties^a^		Internalizing behavior^b^		Externalizing behavior^b^	
	*b*(*SE*)	*t*	*b*(*SE*)	*t*	*b*(*SE*)	*t*
Intercept	25.84 (4.64)***	5.58	39.16 (3.36)***	11.67	48.97 (3.23)-	15.16
Covariates						
Child's gender	−1.65 (1.79)	−0.92	0.48 (1.30)	0.37	0.75 (1.25)	0.60
Education medium	−4.32 (4.52)	−0.96	3.84 (3.28)	1.17	−0.22 (3.16)	−0.07
Education High	**−10.35** (4.33)*	−2.39	1.48 (3.13)	0.47	−2.85 (3.01)	−0.95
Group	0.59 (1.82)	0.31	**3.19** (1.33)*	2.41	1.29 (1.28)	1.01
Mother-child-interaction						
Maternal warmth	2.12 (3.49)	0.61	−0.98 (2.54)	−0.39	1.41 (2.45)	0.58
Maternal stimulation	−0.70 (1.57)	−0.45	0.22 (1.15)	0.19	−0.54 (1.10)	−0.49
Child's negative affect	5.42 (5.42)	1.00	1.16 (3.92)	0.30	3.61 (3.78)	0.96
Dyadic synchrony	**−4.55** (2.03)*	−2.24	−0.33 (1.49)	−0.22	−0.56 (1.43)	−0.39
Active mother and child engagement	0.16 (2.42)	0.07	−1.77 (1.75)	−1.01	−2.65 (1.68)	−1.57
Tense interaction	−2.82 (8.01)	−0.35	−3.12 (5.80)	−0.54	3.03 (5.58)	0.54
Moderation effects						
Maternal warmth*Group	−0.46 (4.87)	−0.09	−1.20 (3.53)	−0.34	−0.89 (3.40)	−0.26
Maternal stimulation*Group	−1.11 (2.08)	−0.54	−0.11 (1.51)	−0.07	−0.18 (1.46)	−0.12
Child's negative affect*Group	−4.72 (7.15)	−0.66	0.38 (5.17)	0.07	−2.49 (4.98)	−0.50
Dyadic synchrony*Group	**6.40** (2.79)*	2.30	1.76 (2.03)	0.87	2.07 (1.95)	1.06
Active mother and child engagement*Group	−0.98 (3.08)	−0.32	1.31 (2.23)	0.59	−0.33 (2.14)	−0.15
Tense interaction*Group	−3.07 (9.88)	−0.31	−1.46 (7.14)	−0.20	−7.71 (6.88)	−1.12
R2	0.12		0.09		0.09	
F	1.57		1.09		1.15	
Conditional effect of Maternal warmth						
Term	2.12 (3.49)	0.61	−0.98 (2.54)	−0.39	1.41 (2.45)	0.58
MLP	1.67 (3.39)	0.49	−2.19 (2.45)	−0.89	0.52 (2.36)	0.22
Conditional effect of Maternal stimulation						
Term	−0.70 (1.57)	−0.45	0.22 (1.15)	0.19	−0.54 (1.10)	−0.49
MLP	−1.81 (1.37)	−1.32	0.10 (0.99)	0.11	−0.72 (0.96)	−0.75
Conditional effect of Child's negative affect						
Term	5.42 (5.42)	1.00	1.16 (3.92)	0.30	3.61 (3.78)	0.96
MLP	0.70 (4.60)	0.15	1.54 (3.32)	0.46	1.12 (3.20)	0.35
Conditional effect of Dyadic synchrony						
Term	**−4.55** (2.03)*	−2.24	−0.33 (1.49)	−0.22	−0.56 (1.43)	−0.39
MLP	1.85 (1.92)	0.97	1.43 (1.39)	1.03	1.51 (1.34)	1.13
Conditional effect of Active mother and child engagement						
Term	0.16 (2.42)	0.07	−1.77 (1.75)	−1.01	−2.65 (1.68)	−1.57
MLP	−0.82 (1.95)	−0.42	−0.46 (1.40)	−0.32	**−2.97** (1.35)*	−2.21
Conditional effect of Tense Interaction						
Term	−2.82 (8.01)	−0.35	−3.12 (5.80)	−0.54	3.03 (5.58)	0.54
MLP	−5.89 (5.74)	−1.02	−4.58 (4.15)	−1.10	−4.68 (4.00)	−1.17

Unstandardized coefficients are presented. Results of three regression analyses are shown, as analyses were performed per outcome measure. Child's gender; 0 = boy, 1 = girl; Group; 0 = term, 1 = Moderate to Late Preterm; MLP = Moderate to Late Preterm.

^a^ Data of one MLP was missing.

^b^ Data of one MLP and two term born children were missing.

* *p *< .05.

** *p *< .01.

*** *p *< .001.

**Table 6:** Results of the regression analyses of mother-child behavior observed in the structured task context, per psychosocial outcome measure.

Structured task context	Social emotional difficulties^a^		Internalizing behavior^b^		Externalizing behavior^b^	
	*b*(*SE*)	*t*	*b*(*SE*)	*t*	*b*(*SE*)	*t*
Intercept	26.88 (4.49)***	5.98	37.86 (3.33)***	11.36	48.21 (3.26)***	14.77
Covariates						
Child's gender	−2.01 (1.72)	−1.17	0.40 (1.28)	0.31	0.43 (1.25)	0.35
Education medium	−6.50 (4.38)	−1.48	5.10 (3.26)	1.57	0.58 (3.19)	0.18
Education High	**−10.44** (4.17)*	−2.50	3.29 (3.09)	1.07	−1.65 (3.02)	−0.55
Group	0.15 (1.76)	0.08	**3.01** (1.31)*	2.28	1.28 (1.29)	1.00
Mother-child interaction						
Maternal warmth	−0.22 (3.67)	−0.06	−0.99 (2.74)	−0.36	−0.50 (2.68)	−0.19
Maternal stimulation	0.90 (1.65)	0.55	1.79 (1.25)	1.43	1.20 (1.22)	0.98
Child's negative affect	3.39 (2.77)	1.22	1.12 (2.10)	0.53	0.55 (2.05)	0.27
Dyadic synchrony	**−5.15** (2.15)*	−2.40	−1.75 (1.68)	−1.04	−0.72 (1.64)	−0.44
Active mother and child engagement	**−4.31** (2.03)*	−2.12	**−3.52** (1.53)*	−2.30	**−3.32** (1.50)*	−2.21
Tense interaction	−7.10 (4.01)	−1.77	−0.97 (3.03)	−0.32	1.33 (2.97)	0.45
Moderation effects						
Maternal warmth*Group	4.65 (5.00)	0.93	−1.16 (3.72)	−0.31	2.23 (3.64)	0.61
Maternal stimulation*Group	−1.06 (2.25)	−0.47	−2.25 (1.69)	−1.33	0.03 (1.66)	0.02
Child's negative affect*Group	−2.56 (3.97)	−0.65	−0.31 (2.98)	−0.11	−0.99 (2.91)	−0.34
Dyadic synchrony*Group	0.78 (2.77)	0.28	1.91 (2.12)	0.90	0.01 (2.08)	<0.01
Active mother and child engagement*Group	2.00 (2.79)	0.72	3.45 (2.09)	1.66	3.25 (2.04)	1.60
Tense interaction*Group	3.54 (5.07)	0.70	<0.01 (3.81)	<.01	0.24 (3.73)	0.07
R2	**0.19** ***		0.12		0.09	
F	2.67		1.51		1.16	
Conditional effect of Maternal warmth						
Term	−0.22 (3.67)	−0.06	−0.99 (2.74)	−0.36	−0.50 (2.68)	−0.19
MLP	4.24 (3.42)	1.29	−2.16 (2.53)	−0.85	1.73 (2.48)	0.70
Conditional effect of Maternal stimulation						
Term	0.90 (1.65)	0.55	1.79 (1.25)	1.43	1.20 (1.22)	0.98
MLP	−0.16 (1.53)	−0.11	−0.45 (1.14)	−0.40	1.23 (1.12)	1.10
Conditional effect of Child's negative affect						
Term	3.39 (2.77)	1.22	1.12 (2.10)	0.53	0.55 (2.05)	0.27
MLP	0.83 (2.82)	0.29	0.81 (2.09)	0.39	−0.44 (2.04)	−0.22
Conditional effect of Dyadic synchrony						
Term	**−5.15** (2.15)*	−2.40	−1.75 (1.68)	−1.04	−0.72 (1.64)	−0.44
MLP	**−4.38** (1.77)*	−2.48	0.15 (1.31)	0.12	−0.72 (1.28)	−0.56
Conditional effect of Active mother and child engagement						
Term	**−4.31** (2.03)*	−2.12	**−3.52** (1.53)*	−2.30	**−3.32** (1.50)*	−2.21
MLP	−2.31 (1.92)	−1.21	−0.07 (1.42)	−0.05	−0.07 (1.39)	−0.05
Conditional effect of Tense interaction						
Term	−7.10 (4.01)	−1.77	−0.97 (3.03)	−0.32	1.33 (2.97)	0.45
MLP	−3.56 (3.10)	−1.15	−0.97 (2.29)	−0.42	1.58 (2.24)	0.70

Unstandardized coefficients are presented. Results of three regression analyses are shown, as analyses were performed per outcome measure. Child's gender; 0 = boy, 1 = girl; Group; 0 = term, 1 = Moderate to Late Preterm; MLP = Moderate to Late Preterm.

^a^ Data of one MLP child was missing.

^b^ Data of one MLP and two term born children were missing.

* *p *< .05.

** *p *< .01.

*** *p *< .001.

#### 3.3.1. Free-play context

The total model of the free-play context explained 12% of the variance in social-emotional difficulties [*R^2^* = .12, *F*(16, 183) = 1.57, *p* = .08], 9% of the variance in internalizing behaviors [*R^2^* = 0.09, *F*(16, 181) = 1.09, *p* = .37] and 9% of the variance in externalizing behaviors [*R^2 ^*= 0.09, *F*(16, 181) = 1.15, *p* = .31]. Even though the models did not significantly explain variance in the psychosocial outcome measures, significant relationships within the models were found.

Regarding the covariates, a high educational level vs. a low educational level, significantly predicted less social-emotional difficulties [*b* = −10.35, *t*(183) = −2.39, *p* = .02]. MLP children showed significantly more internalizing behaviors than term born children, [*b *= 3.19, *t*(181) = 2.41, *p* = .02].

**Figure 1:**
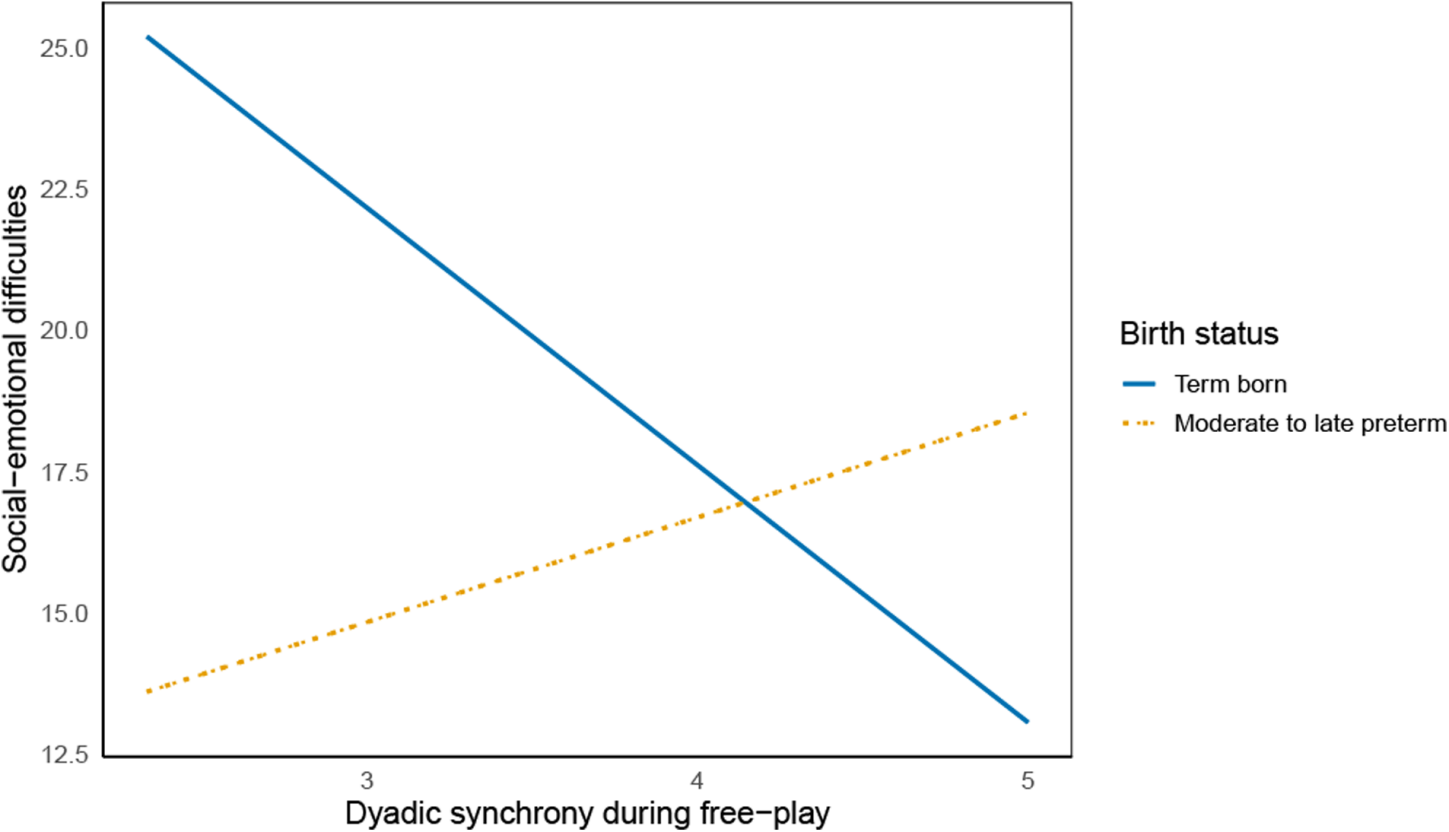
Moderation effect of birth status on the relationship between dyadic synchrony during free-play and children’s social-emotional difficulties.

Regarding the mother-child interaction subscales: Higher levels of dyadic synchrony during free-play significantly predicted less social-emotional difficulties in term born children [*b* = −4.55, *t*(183) = −2.24, *p* = .03], but not in MLP children [*b* = 1.85, *t*(183) = 0.97, *p* = .34]. These relationships were significantly different from one another, as shown by the significant moderation effect of group [*b* = 6.40, *t*(183) = 2.30, *p* = .03]. See Figure 1 for a visual representation of the interaction effect. Furthermore, active mother and child engagement during free-play was related to less externalizing behaviors in the MLP group [*b* = −2,97, *t*(181) = −2.21, *p* = .03], but not in the term born group [*b* = −2,65, *t*(181) = −1.57, *p* = .12]. Nevertheless, birth status did not moderate the association between active mother and child engagement and externalizing behaviors, indicating that the relationships found within the groups were not significantly different between the groups.

#### 3.3.2. Structured task context

The total model of the structured task context explained 19% of the variance for social-emotional difficulties [*R*^2^ = .19, *F*(16, 183) = 2.67, *p* < .001], 12% for internalizing behaviors [*R*^2^ = 0.12, *F*(16, 181) = 1.51, *p* = .10] and 9% for externalizing behaviors [*R*^2^ = 0.09, *F*(16, 181) = 1.16, *p* = .30]. The total model of social-emotional difficulties was significant, but the models for internalizing and externalizing behaviors were not. However, significant relationships within all the models were found.

Regarding the covariates, maternal higher educational level compared to a low educational level, significantly predicted less social-emotional problems, [*b* = −10.44, *t*(183) = −2.50, *p* = .01]. MLP children had significantly more internalizing behaviors [*b* = 3.00, *t*(181) = 2.28, *p* = .02].

Regarding the mother-child interaction subscales, higher levels of dyadic synchrony significantly predicted less social-emotional difficulties in term born children [*b* = −5.15, *t*(183) = −2.40, *p* = .02], as well as in MLP children [*b* = −4.38, *t* (183) = −2.48, *p* = .02]. Additionally, higher levels of active mother and child engagement during the structured task predicted lower levels of social-emotional difficulties [*b* = −4.31, *t*(183) = −2.12, *p* = .04], less internalizing behavior [*b* = −3.52*, t*(181) = −2.30, *p* = .02], and less externalizing behavior [*b* = −3.32, *t*(181) = −2.21, *p* = .03] for children born at term. These relationships were not found for the MLP group (social-emotional difficulties: *b* = −2.31, *t*(183) = −1.21, *p* = .23; internalizing behavior: *b* = −0.07, *t*(181) = −0.05, *p* = .96; externalizing behavior: *b* = −0.07, *t*(181) = −0.05, *p* = .96). Nevertheless, birth status was not a statistically significant moderator between active mother and child engagement and the outcome measures. This indicates that these relationships within each group were not significantly different between the MLP and term born group.

## 4. Discussion

The current study explored differences in the assessment of mother-child interaction in a free-play and a structured task context as well as the associations of these interactive behaviors in each context with psychosocial outcomes in MLP vs. term born toddlers. Our results showed that most mother-child interactive behaviors can be reliably assessed in both observational contexts. Furthermore, differences in mother-child interactive behaviors are observed per context, with the structured task context eliciting a greater variation in behaviors than the free-play context, including more negative and tense interactions as well as more active and engaged behaviors. Lastly, our results showed that the relationship between mother-child interaction and psychosocial outcomes depends on the observational context and the gestational age of the children, reflected in being born MLP vs. term. Associations between mother-child interaction at 18 months of (corrected) age and psychosocial outcomes at 24 months of (corrected) age were mainly found for the structured task context and for dyads with term born children. Active, engaged and synchronous interactive behaviors were associated with better psychosocial outcomes in toddlers, with some differences observed for MLP vs. term born children and for the free-play vs. the structured task context.

 First, we analyzed whether mother-child interaction could be reliably assessed in each observational context. The factor analysis that we used to create subscales of mother-child interaction distinguished six factors that made theoretical sense. However, the subscale of active mother and child engagement combined somewhat unexpected behaviors: Next to positive behaviors, such as child's initiation and child's affection, maternal intrusiveness and mother-led behaviors – behaviors that are typically regarded as negative parenting practices (32) – also loaded positively, though weakly, on this subscale. It should be noted that an important part of the description of maternal intrusiveness and mother-led behaviors concerns re-directing the child's attention (18). These behaviors may therefore – in the current study – be conceptually closer to stimulating and guiding behaviors. Under certain circumstances, these stimulating and guiding behaviors may be needed, e.g., in a context in which the child is presented with a challenging task, and may then be viewed as positive behaviors. More research is needed to test this assumption. Analyses regarding the internal consistency showed satisfactory to good results for the mother-child interaction subscales, indicating that the subscales were measured reliably within and across the free-play and structured task context. Two exceptions were found: tense interaction during free-play and child's negative affect across contexts. Tense interactive behaviors were barely observed during the free-play context which may explain the low internal consistency. Therefore, we advise researchers and practitioners to use a structured task context when their goal is to observe tense interactions. The subscale of the child's negative affect showed good internal consistency in each context separately, but was lower across contexts. This lower internal consistency suggests that the same behaviors used to measure child's negative affect may not represent the same underlying construct in the free-play context vs. the structured task context. In other words, child's negative affect may represent a different construct in each context. However, as child's negative affect was almost non-existent in the free-play context, our data does not allow us to further investigate how the concepts may differ between the contexts. Future studies are needed to draw stronger conclusions regarding the interpretation of child's negative affect in each context. Nevertheless, based on our results, we advise researchers and practitioners not to sum or average child's negative affect across contexts as a combined score may not represent a unidimensional construct. Overall, apart from the lower internal consistency of the tense interaction subscale during free-play, the mother-child interaction subscales were reliably measured in both contexts, justifying further analyses.

Secondly, we compared mother-child interaction between 1) observational contexts, and 2) the MLP and term born group. Our comparison between the two contexts indicated that the structured task context elicits a greater variety of interactive behaviors in both MLP and term born mother-child dyads. More negative behaviors, i.e., child's negative affect and tense interactions, were found which is in line with previous research (6, 13, 14). Additionally, more active and engaged behaviors were found in the structured task context, which is in line with findings of Dittrich and colleagues (2017) in 5–12 year old children. Dittrich and colleagues (2017) explained this finding by suggesting that sharing a mutual goal elicits more functional behaviors, such as responsiveness and emotional availability – a reasoning that may also apply to our findings of more active mother and child engagement during the structured task context. Furthermore, in the MLP group but not in the term born group, dyadic synchrony was lower during the structured task than during the free-play context. This may be explained by the somewhat lower attentional capacities of MLP children (33), which can make it more difficult for the child to focus on the task. In turn, the mother may have to try harder to accomplish the task. The combination of the lower attention span of the child and the mother's attempt to accomplish the task may result in less fluent interactions and less adaptation to each other's levels of involvement on the task, i.e., less dyadic synchronous behaviors. Furthermore, subscales consisting of only maternal behaviors, i.e., maternal warmth and maternal stimulation, were equally observed in both contexts. This is in line with a study of Miller et al., (2002) concluding that mothers show more consistent behaviors across situations, whereas children seem to be more affected by a challenging context, e.g., a structured task context. In the current study, materials for the structured task context were chosen based on the zone of proximal development of 18 months old children, i.e., challenging for the child, explaining why children seemed to be more affected than mothers by the structured tasks. To conclude, the structured task context seems to be a more challenging context for children rather than for mothers, and seems to elicit a greater variety of behaviors including more child's negative affect and tense interactions along with more active and engaged behaviors of both mothers and children. For MLP children, the structured task context may especially be more challenging than the free-play context as dyadic synchrony was lower.

No differences on the mother-child interaction subscales were found between the MLP group and the term born group for both contexts. This is in line with a meta-analysis reporting no differences on maternal sensitivity and responsiveness between preterm, including MLP, and term born mother-child dyads (34). In another meta-analysis, however, was concluded that preterm children, including MLP children, were less alert and more passive and mothers of preterm children more controlling, active and directive (35). A recent study with a similar design as our study – i.e., observing mother-toddlers dyads in a free-play context using the same observation coding scheme as the current study – reported differences in mother-child interaction between preterm (24–34 weeks of GA) and term born mother-child dyads (12). Less maternal sensitivity, child involvement and dyadic reciprocity were found in preterm mother-child dyads compared to the term born group, whereas maternal intrusiveness was higher in the preterm group. However, the differences in mother-child interaction between the two groups depended on the level of prematurity: the differences were larger for very preterm vs. term born children than for moderate preterm vs. term born children (12). In the current study, moderate and also late preterm children were included in the preterm group which may have led to more subtle, non-significant differences between the MLP and term born group. In conclusion, our results indicate that the quality of mother-child interaction is comparable between the MLP and term born mother-child dyads. This can be seen as a positive finding, as all mothers and children show more or less the same interactive behaviors. However, MLP children, as compared to term born children, may need higher levels of specific parental behaviors in order to reach their full potential. Indeed, studies have reported that a higher quality of parent-child interaction, e.g., maternal responsiveness, was more beneficial for social and emotional competences of preterm than term born children in the first year of life (10, 15). However, these studies did not specifically focus on MLP children, nor on the stability of the effects throughout toddlerhood, which may be objectives for future research.

The results of our main research question, i.e., the role of observational context in finding associations with children's psychosocial outcomes, showed that during a structured task as compared to free-play, more associations between mother-child interaction and toddlers' psychosocial outcomes were found. This is in line with the study of Nordahl et al. (2020). The structured task context seems to elicit more meaningful behaviors for finding associations with children's psychosocial outcomes. One of the reasons that could explain this finding is that a structured task forms a more challenging context, which can pressure the interaction between mother and child – as shown by more negative emotions but also more active and engaged behaviors. As such, the structured task may unmask more (dys)functional patterns which are more likely to be related to children's psychosocial outcomes. To illustrate, the stress induced by trying to perform a task together can elicit more negative interaction patterns between mother and child, such as more tense interactions and more negative affect, which are dysfunctional behaviors in view of a stimulating and fun interaction. On the other hand, performing a task together can increase active and engaged behaviors of both parties, i.e., functional behaviors. Therefore, structured tasks especially, may unmask a clear picture of the dynamics between mother and child, revealing more meaningful behaviors in relation to children's psychosocial outcomes.

Furthermore, we investigated whether relationships between mother-child interaction and children's outcomes differed for MLP and term born children. Term born children's outcomes were more often associated with mother-child interaction than MLP children's outcomes. For term born children during a structured task only, active mother and child engagement was related to less social-emotional difficulties, internalizing and externalizing behaviors. For MLP children during a free-play context only, active mother and child engagement was related to less externalizing behaviors. However, the relationships between active mother and child engagement and psychosocial outcomes within both contexts were not moderated by birth status, meaning that the relationships found within the MLP and term born groups were not significantly different from each other. Therefore, it cannot be concluded that different patterns were found between groups for the relationship between active mother and child engagement and psychosocial outcomes. For both MLP and term born children, dyadic synchrony in the structured task was related to less social-emotional problems. Interestingly, in the free-play context, more dyadic synchrony was related to less social-emotional difficulties in the term born group but not in the MLP group. Birth status significantly moderated this relationship, indicating that the relationship between dyadic synchrony during free-play and social-emotional difficulties was significantly different between the groups. It is unclear why more dyadic synchrony during free-play was associated with less-social-emotional difficulties in term born children, but not in MLP children. In addition, it is noteworthy that only within the MLP group – and not in the term born group – lower levels of dyadic synchrony were found for the structured task context than for the free-play context. These two findings are indications that dyadic synchrony may behave differently depending on the population under study (i.e., MLP vs. term born children) as well as the context in which it is observed (i.e., free-play vs. structured task). More research is needed to confirm these results and find reasons for this effect.

Our general finding that mother-child interaction is related to psychosocial outcomes in term born children, but to a lesser extent in MLP children, cannot be explained by differences in mother-child interaction between the groups as all subscales had comparable scores in the MLP and term born group. Nonetheless, when looking into the scores of the subscales, MLP mother-child dyads have consistently, though non-significantly, lower scores on most of the positive mother-child interactions and higher scores on tense interactions. Possibly, there are subtle differences in interactive behaviors between MLP and term born mother-child dyads that explain why MLP children's psychosocial outcomes are to a lesser extent related to mother-child interaction than term born children's psychosocial outcomes. Previous research on the current sample showed that MLP children have less developed receptive communication skills than term born children at 24 months of (corrected) age (36). MLP children may therefore learn less from mother-child interaction. MLP children may need more active, engaged and synchronous mother-child interaction during structured task contexts – the context which seemed to elicit most meaningful interactions in finding associations with psychosocial outcomes in term born children – than term born children in order to reach their full potential. More research is needed to investigate whether increasing active, engaged and synchronous behaviors is beneficial for MLP children and whether this only applies to a structured task context.

For the interpretation of our results, two factors should be considered. First, due to the correlational design of the current study, no causal conclusions can be drawn. Secondly, the effect sizes of the statistical models were rather small. However, as the current study was of exploratory nature, the aim was to identify which mother-child interactive behaviors are important to observe in which context in order to find associations with children's outcomes. Our findings give clear indications of which mother-child interactive behaviors are relevant to observe and in which context. This allows us to provide recommendations for clinical practice as well as researchers.

For both practitioners and researchers, we advise to observe in a structured task when the goal is to elicit a variety of behaviors, including negative and tense interactions as well as more active and engaged behaviors. If the aim is to observe mainly positive emotions, the free-play context is most suitable. Either context can be chosen to observe maternal stimulation and maternal warmth, as these behaviors were equally observed across contexts. For researchers who aim to predict psychosocial outcomes in toddlers based on mother-child interaction, it is advised to study interactive behaviors characterized by active, engaged and synchronous behaviors. For term born toddlers, observing in the structured task context may suffice, whereas for MLP children both contexts seem to be equally relevant. For clinical practitioners who aim to stimulate children's psychosocial development, our findings may suggest targeting interventions to increase mother and child's active and engaged behaviors, as well as dyadic synchronous behaviors. More specifically, for MLP children, the findings suggest targeting interventions to increase dyadic synchronous behaviors during structured task contexts, as well as active and engaged behaviors during free-play. For term born children, the findings suggest targeting interventions to enhance dyadic synchrony in both free-play and structured task contexts, as well as active and engaged behaviors in structured task contexts.

The current study contains strengths as well as limitations. The strengths are that a multi-method is used, i.e., observational measures and parent-report, which gives better insight in the actual behaviors compared to using only parent-report or observations. Second, the current study includes MLP and term born children whereas many studies focus on only extreme to very preterm children or the total range of preterm born children. Since MLP children form 85% of the children born preterm (37), this is an important group to study more in-depth. Limitations may have risen from the design of the current study. The structured task context included two tasks (i.e., puzzle and book) and it would therefore be preferred if the free-play context also consisted of two parts to optimally elicit mother-child interaction. Future research can take a second free-play setting into account, for example with a different selection of toys, or no toys to simply observe how mother and child play with one another. Another limitation to acknowledge is that the mother-child interaction subscales were generated based on a factor analysis, which means that these subscales are dependent on the current sample – which partly consists of an at-risk sample, i.e., MLP children. Moreover, in our study, maternal intrusiveness and mother-led interaction unexpectedly loaded on a positive scale – i.e., active mother and child engagement – that was related to less psychosocial difficulties in children. More research in other samples is needed to investigate whether these subscales can be replicated. It would also be interesting for future research to replicate the current study using a different mother-child interaction coding scheme. Potentially, another coding scheme – such as a micro-coding scheme – would be informative, as this may reveal more information regarding which aspects of – what is generally called – maternal intrusive and leading behaviors can be beneficial for children's psychosocial outcomes and under which circumstances, e.g., in the presence of certain other observed behaviors of the dyad and/or in a specific observational context. Furthermore, a micro-coding scheme may pick up different mother-child interactive behaviors that are relevant for finding associations with children's psychosocial outcomes. Results may confirm our findings, and/or add to our study by providing more insight into the dynamics between mothers and their (pre)term born children in each context in relation to children's psychosocial outcomes.

 In conclusion, the structured task context seems to elicit more meaningful interactions for finding associations with term-born children's psychosocial outcomes than the free-play context. Mother-child interactions characterized by active, engaged and synchronous behaviors were associated with better psychosocial outcomes in toddlers, with some differences observed for MLP vs. term born children and for the free-play vs. the structured task context.

## Data Availability

The data analyzed in this study is subject to the following licenses/restrictions: The data analyzed in this study can be shared upon reasonable request. Requests to access these datasets should be directed to Lisa Krijnen; l.j.g.krijnen@uu.nl.
